# Urbanisation Favours Ground Beetle (Carabidae) Species That Prefer Dry Soils and Have Reduced Dispersal Capacity

**DOI:** 10.1002/ece3.73872

**Published:** 2026-06-21

**Authors:** Jack R. Walker, Karl L. Evans, Rachel M. Jeffreys, Catherine L. Parr

**Affiliations:** ^1^ School of Environmental Sciences University of Liverpool Liverpool UK; ^2^ Ecology and Evolutionary Biology, School of Biosciences University of Sheffield Sheffield UK

**Keywords:** carabid, community‐weighted mean, grassland, replication, trait, urban–rural gradient

## Abstract

Urbanisation imposes strong environmental filters on ecological communities through habitat fragmentation, increased local temperatures, pollution, and the presence of invasive species. Species possessing functional traits that enable them to tolerate these conditions are expected to become more prevalent in highly urbanised areas. However, it remains unclear whether cities with similar climates and regional species pools show comparable trait‐urbanisation relationships. We examined how five functional traits of carabid beetles (Coleoptera: Carabidae) vary along an urban–rural gradient in two climatically and geographically similar UK cities: Liverpool and Manchester. Eighty grassland sites were sampled along an urban–rural gradient in Liverpool and Manchester using pitfall traps. Variation in the community‐weighted mean (CWM) values of traits was analysed along a gradient of percentage impervious surface cover for the two cities independently and combined. We found that CWM latitudinal range, the proportion of macropterous (long‐winged) individuals, and the proportion of individuals of wet soil‐preferring species declined with increasing urbanisation in the combined dataset. Trait responses were not identical across cities: the proportion of individuals of wet soil‐preferring species declined with increasing urbanisation in Manchester only, whereas the proportion of macropterous individuals declined with increasing urbanisation in Liverpool only. Our findings demonstrate that even ecologically similar cities can differ in the trait‐based responses of local beetle assemblages, reinforcing the need for multi‐city sampling in urban ecology. We also highlight that urbanisation can favour species adapted to dry soils and those with reduced dispersal capacity, challenging commonly held assumptions about the advantages of flight in fragmented landscapes.

## Graphical abstract

Species' responses to urbanisation can be associated with their functional traits, with species possessing traits that allow them to reduce or overcome the challenges associated with urbanisation expected to exist in greater densities in urban areas. The aim of this study was to investigate how five functional traits of ground beetles (Coleoptera: Carabidae) vary across an urban–rural gradient in two nearby cities: Liverpool and Manchester, UK. We highlight that (1) urbanisation does not always favour species with a high dispersal capacity, (2) reduced rates of soil water infiltration associated with urban environments can restructure invertebrate assemblages, and (3) urban carabid assemblages in geographically close and ecologically and climatically similar urban centres can exhibit divergent trait values.

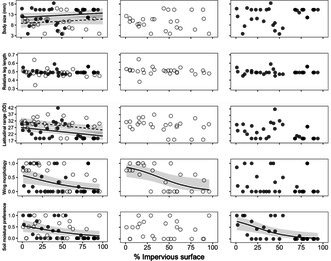

## 1 Introduction

Urban landscapes are one of the fastest growing anthropogenic landforms globally (Seto et al. 2012), presenting a myriad of threats to biodiversity in the form of habitat loss and fragmentation (Cadenasso et al. 2007), elevated temperature via the urban heat island effect (McCarthy et al. 2010), pollution (Villalobos‐Jimenez et al. 2016), and the presence of many non‐native and invasive species (Potgieter et al. 2019). As urban areas expand, it is becoming increasingly important to understand how urban environments filter species according to their functional traits for predicting patterns of biodiversity loss and community restructuring (Evans 2010). A key challenge in urban ecology is determining whether the trait‐based responses observed in one city represent ecological processes that can be generalised to other locations, or are shaped by the historical and environmental context of the study location. Addressing this issue requires studies that compare ecologically similar cities using standardised methods, yet such replication is rare in the urban ecology literature.

Urbanisation acts as an environmental filter, favouring species whose functional traits confer tolerance to this conversion in land use. The replacement of natural habitats with impervious surfaces results in highly fragmented habitats (Cadenasso et al. 2007), increased local temperatures due to the urban heat island effect (McCarthy et al. 2010), and reduced infiltration of rainwater to soil, thereby reducing soil moisture and groundwater replenishment (Kaur et al. 2019). Functional traits, comprising morphological, physiological, or behavioural characteristics that influence fitness, provide a framework for understanding how species respond to the pressures associated with urbanisation (Hahs et al. 2023). Trait‐based approaches can therefore be used to assess which taxa may be likely to persist or decline in urban environments. Across many taxonomic groups (Hahs et al. 2023), including birds (Croci, Butet, and Clergeau 2008; Evans et al. 2009), mammals (Jung and Threlfall 2018; Santini et al. 2018), insects (Magura and Lövei 2021; Ferrari and Polidori 2025), and plants (McKinney 2008; Petersen et al. 2021), urban assemblages are often characterised by species with broad environmental tolerances and traits associated with resilience to disturbance. Urbanisation is also associated with the decline of species with specialist habitat or diet requirements (Concepción et al. 2015; Hahs et al. 2023).

Body size is a particularly important trait as it is related to many other aspects of ecology such as physiology, fecundity, and longevity (Wanninger 2015). Reduced body size is associated with increased urbanisation tolerance in several invertebrate taxa, including Carabidae (Martinson and Raupp 2013), Araneae (Cabon et al. 2024), and Hymenoptera (Eggenberger et al. 2019). This may occur due to urban heat island effects in accordance with the well‐established general pattern of negative temperature‐size responses in ectotherms due to physiological constraints (Atkinson 1994; Verberk et al. 2021). Reduced availability of some foraging resources in urban environments can also favour smaller invertebrates (Ferrari et al. 2024).

High dispersal capacity is often considered to be advantageous for population viability in highly fragmented urban environments, with flight capacity being an important trait for urban insects (Ferrari et al. 2024; Rochat et al. 2017). Here, we discuss dispersal capacity in the context of species that have functional traits that facilitate long‐distance dispersal opportunistically, rather than species with large home ranges that require frequent long‐distance movement between habitat patches. Among invertebrate groups that are ground dwelling and frequently move by means other than flight, other morphological traits can determine capacity to disperse and colonise new patches. Leg length relative to body size, for example, correlates with walking speed in some Coleoptera groups (Barton et al. 2011), and the hind leg to front leg ratio can be an important dispersal factor for jumping insects like Orthoptera (Burrows and Sutton 2008). Contrastingly, some evidence suggests that high dispersal capabilities can be selected against in urban areas of particularly high fragmentation due to the low likelihood of successfully dispersing to suitable habitat (Cheptou et al. 2008; Finand et al. 2023). The impact of urbanisation on dispersal can also act differently depending on spatial scale, with short‐distance, but not long‐distance dispersal being impacted by urbanisation in spiders (Bonte et al. 2023).

Urban‐adapted species may also have a general tolerance for a broad range of environmental conditions (Bonier et al. 2007). Due to large amounts of impervious surface cover reducing infiltration of precipitation into the soil, traits that are adaptive for dry environments can also increase species' tolerance of urban environments (Esperon‐Rodriguez et al. 2020; Menke et al. 2011; Venn et al. 2013; Zhu et al. 2023).

Urbanisation can therefore act as a filter on species traits, influencing which species are able to survive in urban areas (Aronson et al. 2023; Croci, Butet, and Clergeau 2008). This results in urban ecosystems being characterised by reduced taxonomic and functional diversity, and increased similarity among urban assemblages, a process broadly described as biotic homogenisation (McKinney 2002, 2008). An improved understanding of how functional traits of different taxa vary with differing levels of urban intensity (i.e., along an urban–rural gradient) is important for understanding how and why taxa may respond differently to urbanisation.

While broad patterns in trait‐based responses to urbanisation have been detected (McKinney 2008), the majority of studies do not assess multiple urban locations. Therefore, these responses may not be universally predictable. Patterns observed in one city may fail to generalise to others with varying climates, landscape contexts, land‐use history, and regional species pools. Extending the interpretation of results from one urban location to another remains a major challenge in urban ecology.

Ground beetles (Coleoptera: Carabidae) are an ideal taxon for researching trait responses along urban–rural gradients in temperate regions because they are abundant, diverse, and there is marked inter‐specific variation in their tolerance of anthropogenic disturbance (Magura and Lövei 2021). Carabids are well‐represented in the urban traits literature, with a number of studies assessing how species traits are associated with species occurrence patterns in boreal and temperate cities in Asia, Europe and North America (e.g., Croci, Butet, Georges, et al. 2008; Magura et al. 2008; Sadler et al. 2006). For example, species that are capable of flight typically increase in density with increasing urban intensity, while those that are incapable of flight are dominant in rural areas (Perry et al. 2020; Sadler et al. 2006). Similarly, large bodied species are usually found in rural areas, while small bodied species are more abundant in urban areas (Elek and Lövei 2007). Previous research has accumulated evidence of general trends in carabid functional traits among cities, but these trends are by no means universally predictable. Smaller carabids, for example, were found in more urbanised areas in Helsinki (Finland), yet in Edmonton (Canada) there was no trend in body size along the urban–rural gradient (Niemelä et al. 2002). Similarly, Ishitani et al. (2003) found no significant effect of urbanisation on the proportion of small or large species in Hiroshima, Japan.

The aim of this study was to investigate how functional traits of grassland carabids vary across an urban–rural gradient in two nearby cities, Liverpool and Manchester, UK, using identical methods for site selection, sampling and characterising urbanisation gradients. We take a community level approach using a community‐weighted mean (CWM) approach to assessing trait variation (Lavorel et al. 2008). The majority of previous studies examining trait‐urbanisation relationships focus on one city (e.g., Do and Choi 2022; Fournier et al. 2020; Leonard et al. 2018; Magura et al. 2008; Weller and Ganzhorn 2004) or compare cities with different climates (Prass et al. 2017). The GLOBENET Project (Niemelä et al. 2000) assessed the relationship between carabid assemblages and the urban–rural gradient in cities in multiple countries, and helped to address this gap. The associated studies (such as those reviewed in Niemelä and Kotze (2009)), however, implemented a categorical metric of urbanisation (i.e., with urban, suburban, and rural sites) and did not always use a consistent, quantitative method of defining those categories. Thus, despite previous calls for urban research across multiple locations (Evans et al. 2009) there are insufficient studies conducted using identical methods, comparing geographically close cities that share similar environmental conditions and a regional species pool that could occur in all the focal urban areas.

We predict that across both cities as urbanisation intensity increases, carabid assemblages will have a greater proportion of individuals of species with smaller body size, larger relative leg length, broader latitudinal range, large wings, and a preference for dry soils. We focus on grasslands as they are the dominant type of greenspace in temperate cities (Evans et al. 2009), can provide ecologically valuable habitats that support biodiversity within the urban matrix (Helden and Leather 2004; Hemmings et al. 2022), and complement the focus on forest habitats of most urban carabid research (Martinson and Raupp 2013; Niemelä and Kotze 2009).

## 2 Materials and Methods

### 2.1 Site Selection

Field sampling was conducted in June and July 2021 in Liverpool (53.41°N, −2.99°W) and Manchester (53.48°N, −2.24°W), UK (Figure S1). These are the two largest cities in the north‐west of England, with respective estimated populations of 486,100 and 552,000 (ONS 2021), and are approximately 50 km apart when measuring from the urban centre. Manchester was first settled in the 1st century AD as a Roman fort, while Liverpool was founded in the 13th century. Both cities saw rapid urban expansion and population growth during the Industrial Revolution, with Manchester becoming the world's first industrialised city and Liverpool becoming a major port city. Liverpool and Manchester have a very similar climate, with respective mean maximum annual temperatures of 13.57°C and 13.62°C, and mean annual precipitations of 824.32 mm and 868.40 mm (Met Office 2020). The two cities and their surrounding countryside also share a similar regional carabid species pool (Luff 1998). We created a standard metric of urbanisation intensity by calculating the percentage impervious surface of each 500 m × 500 m grid cell (Moll et al. 2019) across the Liverpool and Manchester regions and their surrounding countryside. We visually determined the Liverpool and Manchester urban regions (defined as: Liverpool area bound by the M57 motorway and the River Mersey, plus Maghull and Kirby; Manchester area south of the M60 and M62 motorways) and excluded urban grid cells (defined as ≥ 25% impervious surface cover, following Bonnington et al. (2014)) that were not part of these urban regions to avoid neighbouring urban locations. This was achieved using impervious surface data obtained from the European Environment Agency (2018) in QGIS Geographic Information System. We then selected five focal grid cells within each of eight urbanisation categories in each city: 0%–10%; 11%–23%; 24%–36%; 37%–49%; 50%–62%; 63%–75%; 76%–88%; and 89%–100% impervious surface cover.

Sampling sites were then selected by identifying the closest accessible grassland to the centre of each grid cell, with a minimum size of 374 m^2^ to allow for 15 pitfall traps with 5 m minimum spacing. This resulted in a total of 80 sites (40 sites per city) along an urban–rural gradient of 1%–96% impervious surface cover in Liverpool and 1%–94% in Manchester. However, carabids were only found at 57 sites: 26 in Liverpool and 31 in Manchester. The grasslands included in this study were primarily public parks and incidental, undesignated patches of greenspace, but also included other land uses: rural fields (6), road verges (5), and nature reserves (2).

### 2.2 Carabid Sampling and Identification

At each site, 15 pitfall traps (50 mm diameter; 80 mm depth), containing 40 mL of 1: 1 propylene glycol‐water mixture as a preservative, were arranged in a 5 × 3 grid. They were spaced a minimum of 5 m apart, and up to 10 m apart when sites were large enough. All carabids were identified to species using Forsythe (2000) and Telfer (2016).

Human interference with traps can be a major hazard in urban areas, and pilot data demonstrated that placing traps for multiple days significantly increased the rate of sample loss. Our sampling design thus focused on increasing sampling effort within each category of urbanisation intensity through increasing the number of traps (15) and sites used (10 sites per urbanisation category), rather than the duration (24 h) at which individual traps were used. Sampling was conducted for 3600 trap hours within each of our 8 urbanisation categories. Rarefaction curves demonstrate that only a small number (~4) additional rare species were missed by our sampling approach (Figure S2), although if sampling duration was increased our pilot data indicate that it is highly likely that missing data from increased human interference with our traps would have severely compromised our dataset. Given that the rare species have negligible influence on CWM values (Lavorel et al. 2008), our sampling design provides a robust approach for addressing how urbanisation influences ecological traits of carabid communities.

### 2.3 Species Traits

Species mean body size (mm from the tip of the clypeus to the tip of the elytra) was measured as the mean size of individuals captured at all study sites. At each sampling site, we measured a maximum of six individuals of each species present, randomly selecting six undamaged individuals for measuring. When a species occurred across multiple sites, this resulted in more than six individuals being measured in total across the study. Following Perry et al. (2020), mean relative leg length was calculated (using the same individuals selected for measuring body size) as the combined length of the hind femur and tibia (mm) divided by body size (mm). Latitudinal range (decimal degrees, DD), which we use as an indicator of environmental tolerance as species that occur across a broad latitudinal range can survive in a broad range of climates (Bonier et al. 2007; Dallas and Kramer 2022; Saupe et al. 2019; Zuo et al. 2023), was calculated using the species' native distribution as recorded in the Global Biodiversity Information Facility (GBIF.org 2024). For wing morphology, species were initially categorised as either macropterous (long‐winged), brachypterous (short‐winged) or dimorphic using existing literature (Lindroth 1992; Luff 1998; Turin 2000). We then examined the wing morphology of specimens of our eight dimorphic species collected from pitfall traps. Specimens of the same species did not vary in their wing morphology, and therefore dimorphic species were categorised as either macropterous (*n* = 3) or brachypterous (*n* = 5) based on the wing morphology of all the individuals in our samples. Soil moisture preference (wet or dry) was sourced from literature (Lindroth 1992; Luff 1998; Turin 2000).

### 2.4 Community‐Weighted Mean Trait Values

For each site, the community‐weighted mean (CWM) value of each functional trait was calculated to test for patterns in trait variation along the urban–rural gradient. Multiple studies have shown that, where functional traits are expected to provide an advantage across a given environmental gradient, trait CWM values vary systematically, reflecting the changes in community composition according to the component species' relative fitness in a given environment (Cornwell and Ackerly 2009; Simons et al. 2016; Wright et al. 2004).

CWMs were calculated using the mean of the trait values present at each site, weighted by the relative abundance of each corresponding species at that site. For the two categorical traits, wing morphology and soil moisture preference, macropterous and wet soil‐preferring species were represented by the number one, and brachypterous and dry‐soil preferring species were represented by zero. Sites where no carabids were collected were excluded from analysis because CWM values cannot be calculated for empty assemblages.

### 2.5 Data Analysis

Data were analysed using R (R Core Team 2023). Generalised linear models were used to test the effects of impervious surface cover on the CWMs of body size, relative leg length, latitudinal range (Gaussian error distribution), wing morphology, and soil moisture preference (binomial error distribution). Each model was run for Liverpool and Manchester data separately, and then combined. We did this to assess if results obtained from the pooled dataset would have been detectable if we followed the dominant approach in urban ecology of conducting research in just one urban location. For models using the combined dataset, ‘city’ (i.e., Liverpool or Manchester) and its interaction term with % impervious surface cover were initially included as fixed factors to test for differences in the slopes of the relationships between impervious surface and trait CWMs across the two cities. This interaction term did not have a significant effect on any trait CWM (Table S1), so the models were simplified by removing the interaction term. We thus present the results of a full model that includes ‘city’ and % impervious surface as fixed effects. Covariates were not standardised so that the ecological implications of the results can be more easily interpreted and compared across studies. We calculated Moran's *I* values using the residuals from each model using the “Moran.*I*” function in the “ape” R package (Paradis and Schliep 2019) to test for spatial autocorrelation, for which we found no evidence (Table S2).

In order to estimate the magnitude of phylogenetic signal, whereby closely related species share similar traits, which may lead to statistical non‐independence of data (Felsensein 1985), we tested for differences in each functional trait between carabid genera. For mean body size and mean relative leg length, which did not exhibit normal distributions, Kruskal–Wallis tests were used. Latitudinal range was tested using a one‐way ANOVA, and categorical traits (wing morphology and soil moisture preference) were assessed using Fisher's exact tests. We did not find statistically significant (*α* < 0.05) evidence of phylogenetic signal for any trait, suggesting that our results are robust to non‐independence arising from shared evolutionary history (Tables S3 and S4).

## 3 Results

### 3.1 Summary of Carabids Collected

A total of 275 carabids from 25 species were collected (21 species in Liverpool and 17 species in Manchester) at 57 sites (26 in Liverpool, 31 in Manchester). Nine species were unique to Liverpool and three were unique to Manchester. The most abundant species were *Pterostichus madidus* (92 individuals), *Poecilus cupreus* (34), and *Bembidion properans* (30) (see Table S5 for full data on the abundance of each species collected in each city).

### 3.2 Trait CWMs and Urbanisation Relationships Within Individual Cities

**FIGURE 1:**
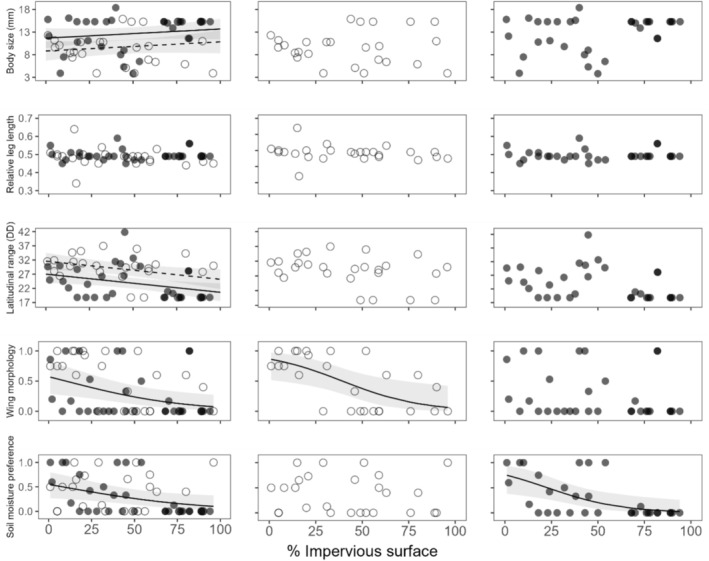
Variation in community‐weighted means (CWM) of body size, relative leg length, latitudinal range, wing morphology, and soil moisture preference along a gradient of % impervious surface cover in models that pool data across cities, and models constructed separately for each city. Points show the CWM for each sampling site (open points represent Liverpool, filled points represent Manchester). Lines show predicted values (and shading the associated 95% confidence intervals) from generalised linear models where effects of % impervious surface were significant (*α* < 0.05; Tables 1 and 2). Panels with two lines indicate where the mean trait CWMst values for Liverpool and Manchester were significantly different to each other (Table 2), with dotted lines representing Liverpool and solid lines representing Manchester. CWM body size did not vary significantly across the urban gradient, but mean values did differ between cities. Relative leg length is presented as a ratio of leg length (mm) to body length (mm). For wing morphology 0 = brachypterous and 1 = macropterous; for soil moisture preference 0 = dry and 1 = wet.

When examining trait patterns in the two cities separately, CWM body size, CWM relative leg length and CWM latitudinal range did not show any significant relationship with impervious surface cover in either city, although in both cities species with broader latitudinal distribution ranges were less common in more urban sites although with only marginal significance (Figure 1 and Table 1). In Manchester, the proportion of individuals of wet soil‐preferring species decreased with increasing impervious surface cover; no significant trend for this trait was found in Liverpool. The proportion of macropterous individuals decreased with increasing impervious surface cover in Liverpool, with no significant trend in Manchester (Figure 1 and Table 1).

**TABLE 1:** Results of generalised linear models of community‐weighted mean trait values in carabid assemblages as a function of % impervious surface cover. Cities (Liverpool and Manchester) were modelled separately. Bold *p*‐values indicate significant (*α* < 0.05) effects of % impervious surface cover.

Trait	City	Predictor	Estimate ± std. error	*R* ^2^	*p*
Mean body size (mm)	Liverpool	Intercept	9.644 ± 1.349	2.906 × 10^−5^	**2.** **160** **×** **10^−7^**
		% impervious surface	0.001 ± 0.026		0.972
	Manchester	Intercept	10.937 ± 1.352	0.067	**4.990** **×** **10^−9^**
		% impervious surface	0.035 ± 0.024		0.152
Mean Relative leg length	Liverpool	Intercept	0.497 ± 0.017	0.034	**< 2.00** **×** **10^−16^**
		% impervious surface	−3.204 × 10^−4^ ± 3.409 × 10^−4^		0.357
	Manchester	Intercept	0.492 ± 0.011	0.003	**< 2.00** **×** **10^−16^**
		% impervious surface	6.105 × 10^−5^ ± 1.903 × 10^−4^		0.751
Latitudinal range (DD)	Liverpool	Intercept	31.623 ± 1.763	0.127	**2.090** **×** **10^−15^**
		% impervious surface	−0.064 ± 0.035		0.075
	Manchester	Intercept	26.999 ± 1.953	0.102	**1.500** × **10^−14^**
		% impervious surface	−0.064 ± 0.034		0.074
Wing morphology	Liverpool	Intercept	1.873 ± 0.914	0.331	**0.040**
		% impervious surface	−0.048 ± 0.020		**0.017**
	Manchester	Intercept	−0.236 ± 0.739	0.044	0.749
		% impervious surface	−0.016 ± 0.014		0.262
Soil moisture preference	Liverpool	Intercept	−0.299 ± 0.728	0.005	0.681
		% impervious surface	−0.005 ± 0.015		0.743
	Manchester	Intercept	1.145 ± 0.823	0.321	0.165
		% impervious surface	−0.050 ± 0.020		**0.012**

### 3.3 Trait CWMs and Urbanisation Relationships in the Combined Dataset

When analysing the combined dataset (i.e., pooling Liverpool and Manchester data), CWM latitudinal range, the proportion of macropterous individuals and the proportion of individuals of wet soil‐preferring species significantly decreased with increasing impervious surface cover (Figure 1 and Table 2). We also found significant differences in the overall CWM mean body size and CWM latitudinal range between Liverpool and Manchester, with Liverpool carabid communities being composed of species that are smaller in body size and have broader latitudinal ranges than those in Manchester (Figure 1 and Table 2).

**TABLE 2:** Results of generalised linear models of community‐weighted mean trait values in carabid assemblages as a function of % impervious surface cover and city (Liverpool and Manchester) in pooled datasets. Bold *p*‐values indicate significant (*α* < 0.05) effects of fixed effects.

Trait	Predictor	Estimate ± std. error	*R* ^2^	*p*
Mean body size (mm)	Intercept	8.825 ± 1.066	0.152	3.040 × 10^−11^
	% impervious surface	0.020 ± 0.018		0.259
	City (Manchester)	2.837 ± 1.020		**0.007**
Mean relative leg length	Intercept	0.488 ± 0.011	0.033	< 2.00 × 10^−16^
	% impervious surface	−1.050 × 10^−4^ ± 1.841 × 10^−4^		0.571
	City (Manchester)	0.014 ± 0.011		0.193
Latitudinal range (DD)	Intercept	31.605 ± 1.466	0.264	< 2.00 × 10^−16^
	% impervious surface	−0.064 ± 0.024		**0.011**
	City (Manchester)	−4.590 ± 1.403		**0.002**
Wing morphology	Intercept	1.137 ± 0.628	0.180	0.071
	% impervious surface	−0.029 ± 0.011		**0.011**
	City (Manchester)	−0.842 ± 0.598		0.159
Soil moisture preference	Intercept	0.497 ± 0.596	0.124	0.404
	% impervious surface	−0.025 ± 0.011		**0.025**
	City (Manchester)	−0.291 ± 0.592		0.623

## 4 Discussion

In the combined dataset, we found no evidence that relationships between trait CWMs and urbanisation intensity vary across cities, despite Liverpool carabid assemblage comprising smaller species with broader latitudinal ranges than those in Manchester. We found that, in Liverpool and Manchester, urbanisation is filtering out species with broader latitudinal ranges, macropterous species (i.e., those with the greatest dispersal capability), and species that prefer wet soils. Yet, if we had only sampled one of the two cities, we would have concluded that these relationships were either only marginally significant (latitudinal range), or they varied across cities (wing morphology and soil moisture preference). Replication is a fundamentally important aspect of ecological research that can improve confidence in field‐based ecological research (Filazzola and Cahill Jr 2021) and our results provide further evidence of the need for multiple cities to be included in urban ecology studies.

Findings from the pooled data set suggest that the proportion of macropterous individuals significantly decreased with increasing % impervious surface; in other words, carabid assemblages in more urbanised settings were dominated by individuals from flightless (brachypterous) species. This result contrasts with previous studies, which suggest that macropterous species are typically found in greater densities in urban areas due to their ability to move within fragmented landscapes and colonise new patches more easily (Niemelä and Kotze 2009). However, an emerging body of urban ecology literature indicates that urbanisation can also impose strong selection pressure against dispersal. In highly fragmented landscapes, dispersal may be detrimental if individuals are likely to disperse to unsuitable habitats, reducing survival or reproductive success. For example, Finand et al. (2023) found that urbanisation favoured wingless queens in the ant *Myrmecina graminicola* due to the increased risk that dispersal does not result in finding suitable habitat patches in highly fragmented locations where such patches are rare. Selection for reduced dispersal capacity may therefore increase fitness by increasing the likelihood of individuals remaining within a known suitable habitat patch, rather than risking dispersal into hostile urban matrices.

This mechanism may be particularly relevant for the focal cities in our study, which have a long history of urban development and consequently exhibit extensive habitat fragmentation. In such landscapes, the distances between suitable habitat patches may exceed the effective range at which the benefits of dispersal outweigh the risks. Contrasting evidence for associations between dispersal capacity and urbanisation intensity may therefore reflect context dependency, driven by the substantial variation in both the magnitude of habitat fragmentation which occurs across urban study locations and the dispersal capacities of different taxa (Liu et al. 2016). These findings suggest that urbanisation does not exert uniform selection pressure on dispersal traits; instead, the direction and magnitude of selection are likely to depend on the interaction between landscape structure and species‐specific dispersal capacities and strategies.

Furthermore, isolated carabid communities have been shown to vary in their flight capabilities depending on how recently the habitat was colonised, with pioneer communities (i.e., the first communities to colonise a new habitat) consisting of mostly macropterous species and macropterous individuals of dimorphic species (den Boer et al. 1980; Venn 2016). Brachypterous individuals of dimorphic species may then become more common over time as they are thought to be able to invest more resources into reproduction, providing a selective advantage when dispersal is less beneficial (Kotze and O'Hara 2003). Therefore, it is perhaps notable that of the eight dimorphic species within our samples (from sites that have typically been urbanised for many decades and in some cases over a century), we find a slightly greater proportion of species that are consistently brachypterous within our assemblages than are macropterous. Over time, brachypterous individuals may increase in frequency as selection shifts from colonisation ability to reproductive investment.

We found no trend in CWM relative leg length along the urban–rural gradient in the combined model. Relative leg length is associated with walking speed in carabids (Fountain‐Jones et al. 2015) and is the only trait relevant for dispersal and patch colonisation for brachypterous carabid species (seven of the total 25 species found in this study). Despite being a potentially important trait for dispersal, relative leg length is rarely included in similar studies of trait‐urbanisation patterns in carabids. This may be because the majority of these studies focus on woodlands, and relative leg length may be a more important trait for insects in open grassland habitats (Kaspari and Weiser 1999). Additionally, urban trait‐based studies often use traits that are associated with filtering at landscape scales (e.g., flight capability), whereas leg length may be more relevant at smaller spatial scales of movement. However, in their study of intraspecific trait variation in carabids, Papp et al. (2020) found that leg length was greatest in either urban or suburban areas for all species studied, relative to rural areas. The contrasting pattern between our CWM‐based results and the intraspecific trends found by Papp et al. (2020) may reflect the differences in the ecological scale at which trait responses are expressed. CWMs integrate shifts in species composition, masking intraspecific patterns in relative leg length that may have been present. The lack of patterns in CWM relative leg length in our study, combined with the significant decrease in the proportion of macropterous individuals with increasing impervious surface cover, indicates that, in our study system, wing morphology is a more important trait for predicting carabid species' responses to urbanisation than relative leg length. Even though relative leg length is expected to be greater for insects in planar habitats such as grasslands (Kaspari and Weiser 1999), macroptery has also been shown to be a highly prevalent trait in urban grassland carabid communities (Venn et al. 2013), further suggesting that flight may be the more important form of dispersal for urban carabids. It is plausible that the ability to disperse long distances across a hostile habitat matrix favours flight over walking speed, reducing the selective advantage of longer legs at the community level.

We expected CWM body size to be negatively associated with urban intensity, yet found no trend in this trait along the urban–rural gradient. We also found that, across the gradient, the Manchester carabid community was composed of larger species than that in Liverpool, and in particular was dominated by *Pterostichus madidus*. The four largest species (*Pterostichus niger*, *P. aethiops* , *P. melanarius* , and *P. madidus* ) were all more common in Manchester than they were in Liverpool, with these species totalling 102 in Manchester and 21 in Liverpool. In contrast, *Bembidion properans* was four times as common in Liverpool than in Manchester and was the third smallest species found in this study. We thus find a marked disparity between cities in the overall abundance of species at the opposite ends of the body size continuum. Body size is an important trait for a variety of functions (Wanninger 2015) and is generally found to decrease with increasing urban intensity in multiple invertebrate taxa (Cabon et al. 2024; Eggenberger et al. 2019), including carabids (Alaruikka et al. 2002; Magura et al. 2004; Sadler et al. 2006). However, there is evidence that the association between small species and high urban intensity only exists for habitat specialists (Magura et al. 2020), and all of the species collected in this study are considered to be habitat generalists (Turin 2000). The lack of variation in CWM body size along the urban–rural gradient in this study may be a product of the fact that we sampled a more open habitat type than the forests that are typically used in studies of urban carabids and did not find any habitat specialists.

We used CWM latitudinal range as a measure of environmental tolerance, predicting that urban carabid communities would comprise species with broader latitudinal ranges due to their occurrence across a broader range of climates. However, our findings contradict this prediction, with CWM latitudinal range decreasing with increasing urban intensity. While the range in species latitudinal range within our dataset is substantial (18.803–41.796 DD), the species with the narrowest latitudinal range (*Pterostichus madidus*) occurs from northern Scotland to northern Spain (GBIF.org 2024) and so is still likely to tolerate a reasonable breadth of environmental conditions. The majority of species occur across the entire latitude of mainland Europe, suggesting that they have broad physiological tolerances which are typical of temperate invertebrate species (Kellermann et al. 2012). It is thus plausible that a stronger signal of specialisation, as measured by range size, would be detected in studies conducted in other regions. The lack of species with narrow latitudinal ranges, combined with the lack of habitat specialists, may contribute to our findings and arguably does not contradict the typical finding that urbanisation favours generalist species. Additionally, our study region is highly modified by both urbanisation and intensive agriculture, therefore lacking some of the more specialist members of the UK carabid assemblage. Consequently, different patterns may emerge if more natural habitats were present on the edges of our focal urban region.

We predicted the proportion of individuals of wet soil‐preferring carabid species would decline with increasing urbanisation, due to impervious surfaces typically leading to drier soil conditions with reduced infiltration of water into soil. Our findings support this prediction. In particular, urban Manchester sites were dominated by *Pterostichus madidus*, a dry soil‐preferring species, which accounted for 80% of carabids collected at sites above 50% impervious surface cover and was the only species found at 69% of those sites. Urbanisation has been found to select for xerophilic species (Esperon‐Rodriguez et al. 2020; Menke et al. 2011; Zhu et al. 2023) and this study provides further evidence of this. Further, it is interesting that we find this effect of urbanisation favouring dry‐tolerant species despite our focal cities receiving relatively high annual rainfall (Liverpool = 824 mm; Manchester = 868 mm; Met Office 2020).

Managing urban greenspaces for biodiversity requires consideration of trait‐based filtering processes. The decline of individuals of wet soil‐preferring species with urbanisation highlights the importance of preserving or restoring microhabitats with higher soil moisture, even in cities with high rainfall. Additionally, the divergent trait patterns between two similar cities underscore the value of multi‐city sampling, as conservation strategies based on single‐city studies may not generalise.

### 4.1 Conclusion

We use a community‐weighted mean approach to assess how urbanisation intensity, measured by percentage impervious surface cover, is associated with species' traits in grassland carabid assemblages across two large urban centres in north‐west England. Our study provides three important findings. First, it highlights that contrary to the dominant view in urban ecology literature, urbanisation does not always select against species with a low dispersal capacity. We draw attention to the need to consider the extent of habitat fragmentation in focal study locations when assessing links between dispersal capacity and urbanisation. Second, we highlight that even in non‐arid environments, urban environments can restructure invertebrate assemblages in favour of species adapted to drier conditions. Finally, we highlight that urban carabid assemblages in geographically close and ecologically and climatically similar urban centres can exhibit divergent trait values (mean body size and latitudinal range) and that studies of urban‐trait associations that are not replicated in more than one urban location may yield unreliable results.

## Author Contributions

**Jack R. Walker:** conceptualization (equal), data curation (lead), formal analysis (lead), investigation (lead), methodology (lead), project administration (lead), visualization (lead), writing – original draft (lead). **Karl L. Evans:** conceptualization (equal), formal analysis (supporting), funding acquisition (equal), methodology (supporting), supervision (supporting), writing – review and editing (equal). **Rachel M. Jeffreys:** conceptualization (equal), formal analysis (supporting), funding acquisition (equal), methodology (supporting), supervision (supporting), writing – review and editing (equal). **Catherine L. Parr:** conceptualization (equal), formal analysis (supporting), funding acquisition (equal), methodology (supporting), supervision (lead), writing – review and editing (equal).

## Funding

This work was supported by the Natural Environment Research Council, NE/S00713X/1.

## Conflicts of Interest

The authors declare no conflicts of interest.

## Supporting information


**Figure S1:** Map of the (a) Liverpool and (b) Manchester areas, with the sampling region defined by the gridded area (cells are 500 m × 500 m). Sampling sites were grasslands selected via random stratified sampling along an urban–rural gradient based on % impervious surface cover.
**Figure S2:** Rarefaction curve showing the relationship between the number of individual carabids collected and the predicted species richness. Approximately four additional species may have been found if the number of individuals collected had been doubled.
**Table S1:** Results of general linear models of community‐weighted mean trait values in carabid assemblages as a function of % impervious surface cover, city (Liverpool and Manchester), and the interaction term between % impervious surface cover and city. Bold *p‐*values indicate significant (*α* < 0.05) effects of fixed effects.
**Table S2:** Moran's *I* values calculated using the residuals of each model.
**Table S3:** Results of Kruskal–Wallis tests (mean body size, mean relative leg length) and Fisher's exact tests (wing morphology, soil moisture preference) used to test for differences in each trait between carabid genera. These tests were used to estimate the magnitude of phylogenetic signal.
**Table S4:** Results of one‐way ANOVA used to test for differences in latitudinal range between carabid genera. This test was used to estimate the magnitude of phylogenetic signal.
**Table S5:** Abundance and trait values of each carabid species collected. Wing morphology is categorised as macropterous (m) or brachypterous (b) and soil moisture preference is categoised as wet (w) or dry (d). Asterisks (*) indicate wing dimorphic species that were categorised as either macropterous or brachypterous based on the wing morphology of the individuals in our samples.

## Data Availability

Data are stored at https://github.com/jackrwalker/carabid2024.
