# Supplementary material for: Urbanisation Favours Ground Beetle (Carabidae) Species That Prefer Dry Soils and Have Reduced Dispersal Capacity
Source: Ecol Evol. 2026 Jun 21;16(6):e73872. doi: 10.1002/ece3.73872 (PMC13284495; doi:10.1002/ece3.73872)
Supplement: Supplementary file 1 — Figure S1: Map of the (a) Liverpool and (b) Manchester areas, with the sampling region defined by the gridded area (cells are 500 m × 500 m). Sampling sites were grasslands selected via random stratified sampling along an urban–rural gradient based on % impervious surface cover. Figure S2: Rarefaction curve showing the relationship between the number of individual carabids collected and the predicted species richness. Approximately four additional species may have been found if the number of individuals collected had been doubled. Table S1: Results of general linear models of community‐weighted mean trait values in carabid assemblages as a function of % impervious surface cover, city (Liverpool and Manchester), and the interaction term between % impervious surface cover and city. Bold p‐values indicate significant (α < 0.05) effects of fixed effects. Table S2: Moran's I values calculated using the residuals of each model. Table S3: Results of Kruskal–Wallis tests (mean body size, mean relative leg length) and Fisher's exact tests (wing morphology, soil moisture preference) used to test for differences in each trait between carabid genera. These tests were used to estimate the magnitude of phylogenetic signal. Table S4: Results of one‐way ANOVA used to test for differences in latitudinal range between carabid genera. This test was used to estimate the magnitude of phylogenetic signal. Table S5: Abundance and trait values of each carabid species collected. Wing morphology is categorised as macropterous (m) or brachypterous (b) and soil moisture preference is categoised as wet (w) or dry (d). Asterisks (*) indicate wing dimorphic species that were categorised as either macropterous or brachypterous based on the wing morphology of the individuals in our samples. [file ECE3-16-e73872-s001.docx]

Supplementary material


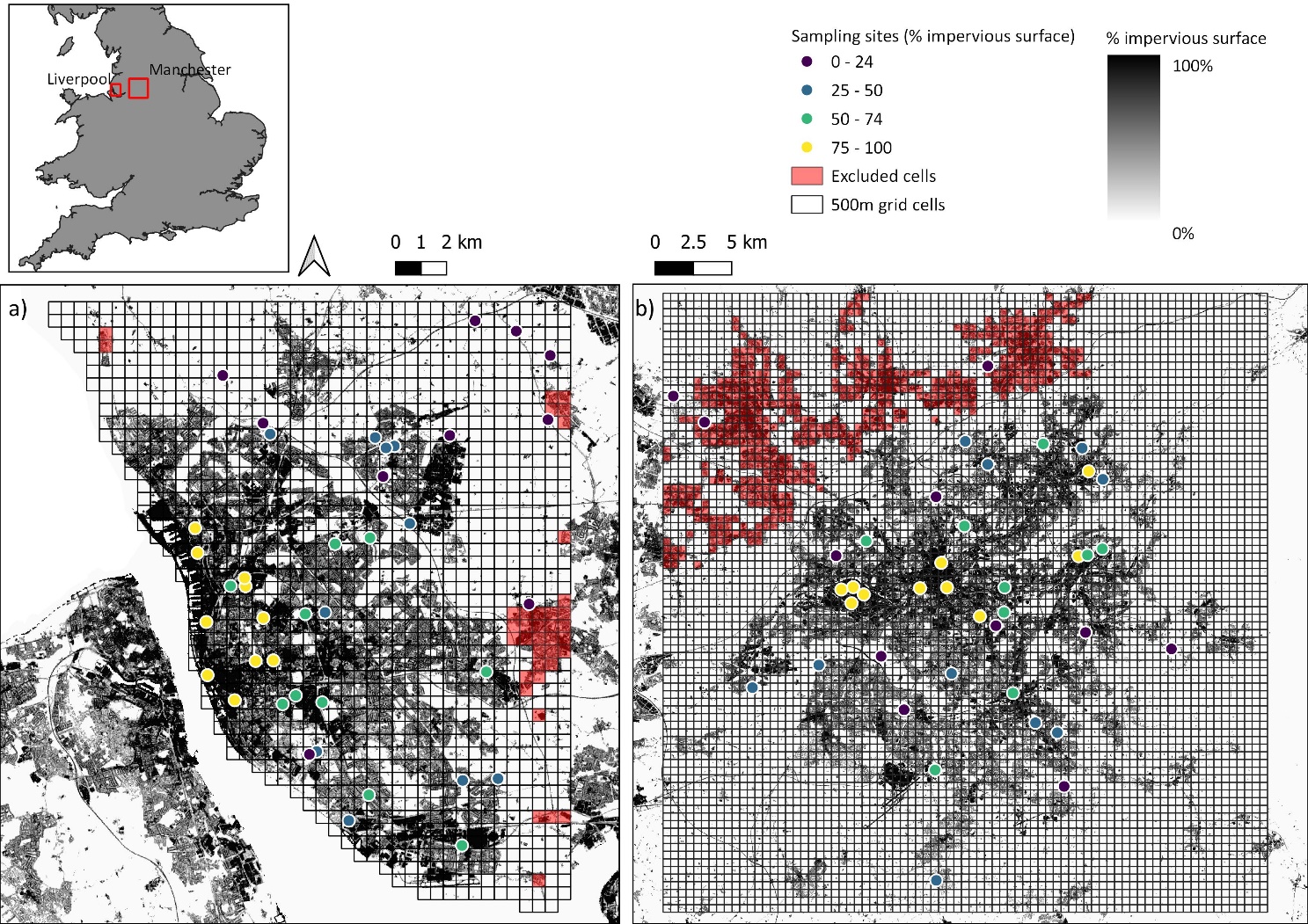


Figure S1. Map of the a) Liverpool and b) Manchester areas, with the sampling region defined by the gridded area (cells are 500 m x 500 m). Sampling sites were grasslands selected via random stratified sampling along an urban-rural gradient based on % impervious surface cover.


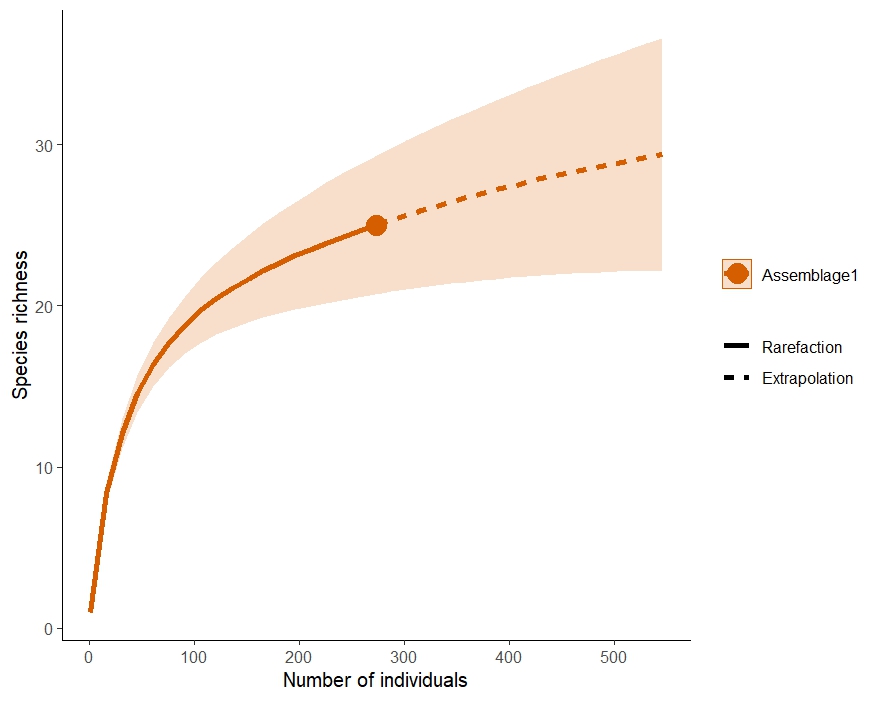


Figure S2. Rarefaction curve showing the relationship between the number of individual carabids collected and the predicted species richness. Approximately four additional species may have been found if the number of individuals collected had been doubled.

Table S1. Results of general linear models of community-weighted mean trait values in carabid assemblages as a function of % impervious surface cover, city (Liverpool and Manchester), and the interaction term between % impervious surface cover and city. Bold *p-*values indicate significant (α < 0.05) effects of fixed effects.

| **Trait** | **Predictor** | **Estimate ± std. error** | **R^2^** | ***p*-value** |
| --- | --- | --- | --- | --- |
| Mean Body size (mm) | Intercept | 9.644 ± 1.370 | 0.166 | 3.500x10^-9^ |
|  | % impervious surface | 0.001 ± 0.027 |  | 0.972 |
|  | City (Manchester) | 1.293 ±1.913 |  | 0.502 |
|  | % impervious surface : City (Manchester) | 0.034 ± 0.036 |  | 0.344 |
| Mean Relative leg length | Intercept | 0.497 ± 0.014 | 0.055 | <2.00x10^-16^ |
|  | % impervious surface | -3.204x10^-4^ ± 2.788x10^-4^ |  | 0.256 |
|  | City (Manchester) | -0.003 ± 0.020 |  | 0.869 |
|  | % Impervious surface : City (Manchester) | 3.814x10^-4^ ± 3.711x10^-4^ |  | 0.309 |
| Latitudinal range (DD) | Intercept | 31.623 ± 1.899 | 0.264 | <2.00x10^-16^ |
|  | % impervious surface | -0.064 ± 0.037 |  | 0.089 |
|  | City (Manchester) | -4.623 ± 2.653 |  | 0.087 |
|  | % impervious surface : City (Manchester) | -0.001 ± 0.050 |  | 0.988 |
| Wing morphology | Intercept | 1.873 ± 0.914 | 0.211 | 0.040 |
|  | % impervious surface | -0.048 ± 0.020 |  | **0.017** |
|  | City (Manchester) | -2.109 ± 1.175 |  | 0.073 |
|  | % Impervious surface : City (Manchester) | 0.031 ± 0.025 |  | 0.201 |
| Soil moisture preference | Intercept | -0.299 ± 0.728 | 0.201 | 0.681 |
|  | % impervious surface | -0.005 ± 0.015 |  | 0.743 |
|  | City (Manchester) | 1.444 ± 1.099 |  | 0.189 |
|  | % Impervious surface : City (Manchester) | -0.045 ± 0.025 |  | 0.067 |

Table S2. Moran’s I values calculated using the residuals of each model.

| Model | Expected | Std. deviation | *p*-value |
| --- | --- | --- | --- |
| Combined body size ~ % impervious surface * city | -0.018 | 0.038 | 0.153 |
| Combined body size ~ % impervious surface + city | -0.018 | 0.038 | 0.174 |
| Liverpool body size ~ % impervious surface | -0.040 | 0.060 | 0.115 |
| Manchester body size ~ % impervious surface | -0.032 | 0.043 | 0.930 |
| Combined relative leg length ~ % impervious surface * city | -0.018 | 0.035 | 0.243 |
| Combined relative leg length ~ % impervious surface + city | -0.018 | 0.035 | 0.194 |
| Liverpool relative leg length ~ % impervious surface | -0.040 | 0.051 | 0.435 |
| Manchester relative leg length ~ % impervious surface | -0.032 | 0.041 | 0.739 |
| Combined latitudinal range ~ % impervious surface * city | -0.018 | 0.037 | 0.709 |
| Combined latitudinal range ~ % impervious surface + city | -0.018 | 0.037 | 0.708 |
| Liverpool latitudinal range ~ % impervious surface | -0.040 | 0.059 | 0.166 |
| Manchester latitudinal range ~ % impervious surface | -0.032 | 0.041 | 0.739 |
| Combined wing morphology ~ % impervious surface * city | -0.018 | 0.038 | 0.976 |
| Combined wing morphology ~ % impervious surface + city | -0.018 | 0.038 | 0.951 |
| Liverpool wing morphology ~ % impervious surface | 0.040 | 0.061 | 0.544 |
| Manchester wing morphology ~ % impervious surface | -0.032 | 0.043 | 0.974 |
| Combined soil moisture preference ~ % impervious surface * city | -0.018 | 0.038 | 0.091 |
| Combined soil moisture preference ~ % impervious surface + city | -0.018 | 0.038 | 0.160 |
| Liverpool soil moisture preference ~ % impervious surface | -0.040 | 0.060 | 0.434 |
| Manchester soil moisture preference ~ % impervious surface | -0.032 | 0.042 | 0.146 |

Table S3. Results of Kruskal-Wallis tests (mean body size, mean relative leg length) and Fisher’s exact tests (wing morphology, soil moisture preference) used to test for differences in each trait between carabid genera. These tests were used to estimate the magnitude of phylogenetic signal.

| **Trait** | **Kruskal-Wallis chi-squared** | ***p*-value** |
| --- | --- | --- |
| Mean body size | 16.529 | 0.085 |
| Mean relative leg length | 14.800 | 0.140 |
| Wing morphology | *NA* | 0.096 |
| Soil moisture preference | *NA* | 0.347 |

Table S4. Results of one-way ANOVA used to test for differences in latitudinal range between carabid genera. This test was used to estimate the magnitude of phylogenetic signal.

|  | Sum of squares | df | Mean squares | F | *p*-value |
| --- | --- | --- | --- | --- | --- |
| Genus | 222.700 | 10 | 22.170 | 0.663 | 0.740 |
| Residuals | 470.100 | 14 | 33.580 | *NA* | *NA* |

Table S5. Abundance and trait values of each carabid species collected. Wing morphology is categorised as macropterous (m) or brachypterous (b) and soil moisture preference is categoised as wet (w) or dry (d). Asterisks (*) indicate wing dimorphic species that were categorised as either macropterous or brachypterous based on the wing morphology of the individuals in our samples.

| **Species** | **Abundance in Liverpool** | **Abundance in Manchester** | **Mean body size (mm) ± std. deviation** | **Median body size (mm)** | **Mean relative leg length ± std deviation** | **Median relative leg length** | **Latitudinal range (DD)** | **Wing morphology (m/b)** | **Soil moisture preference (w/d)** |
| --- | --- | --- | --- | --- | --- | --- | --- | --- | --- |
| *Agonum muelleri* (Herbst, 1784) | 4 | 2 | 7.96 ± 0.29 | 7.98 | 0.53 ± 0.03 | 0.52 | 30.701 | m | d |
| *Amara aenea* (De Geer, 1774) | 11 | 0 | 7.15 ± 0.30 | 7.14 | 0.44 ± 0.04 | 0.44 | 37.199 | m | d |
| *Amara aulica* (Panzer, 1796) | 1 | 0 | 13.00 | 13.0 | 0.48 | 13.0 | 26.362 | m | w |
| *Amara communis* (Panzer, 1797) | 6 | 2 | 7.25 ± 0.34 | 7.28 | 0.47 ± 0.04 | 0.48 | 31.682 | m | w |
| *Amara lunicollis* (Schiödte, 1837) | 6 | 0 | 8.47 ± 0.40 | 8.33 | 0.46 ± 0.03 | 0.46 | 27.603 | m | w |
| *Amara ovata* (Fabricius, 1792) | 4 | 0 | 9.69 ± 0.52 | 9.59 | 0.49 ± 0.01 | 0.48 | 28.090 | m | d |
| *Amara plebeja* (Gyllenhal, 1810) | 5 | 4 | 7.47 ± 0.31 | 7.42 | 0.47 ± 0.01 | 0.48 | 24.476 | m | w |
| *Anchomenus dorsalis* (Pontoppidan, 1763) | 1 | 0 | 7.10 | 7.10 | 0.59 | 0.59 | 27.216 | m | d |
| *Bembidion guttula* (Fabricius, 1792) | 1 | 2 | 3.69 ± 0.32 | 3.50 | 0.48 ± 0.04 | 0.48 | 27.490 | m* | w |
| *Bembidion lampros* (Herbst, 1784) | 6 | 1 | 3.76 ± 0.19 | 3.85 | 0.47 ± 0.04 | 0.47 | 32.656 | b* | d |
| *Bembidion properans* (Stephens, 1828) | 24 | 6 | 3.94 ± 0.79 | 4.20 | 0.45 ± 0.03 | 0.45 | 29.990 | b* | w |
| *Bradycellus harpalinus* (Audinet-Serville, 1821) | 2 | 0 | 4.90 ± 0.40 | 4.90 | 0.37 ± 0.03 | 0.37 | 23.192 | b* | d |
| *Harpalus rufipes* (De Geer, 1774) | 2 | 4 | 13.60 ± 1.57 | 14.35 | 0.49 ± 0.03 | 0.50 | 31.375 | m | d |
| *Harpalus tardus* (Panzer, 1796) | 1 | 0 | 10.20 | 10.20 | 0.45 | 0.45 | 27.004 | m | d |
| *Loricera pilicornis* (Fabricius, 1775) | 1 | 0 | 7.40 | 7.40 | 0.64 | 0.64 | 34.612 | m | w |
| *Nebria brevicollis* (Fabricius, 1792) | 9 | 1 | 11.61 ± 0.62 | 11.76 | 0.56 ± 0.03 | 0.56 | 28.145 | m | d |
| *Notiophilus biguttatus* (Fabricius, 1779) | 0 | 1 | 5.30 | 5.30 | 0.45 | 0.45 | 41.796 | m* | w |
| *Notiophilus rufipes* (Cutris, 1829) | 0 | 1 | 5.20 | 5.20 | 0.54 | 0.54 | 19.463 | m | w |
| *Poecilus cupreus* (Linnaeus, 1758) | 34 | 0 | 10.90 ± 0.51 | 10.92 | 0.51 ± 0.03 | 0.50 | 36.947 | m | d |
| *Pterostichus aethiops* (Panzer, 1796) | 0 | 2 | 16.30 ± 0.10 | 16.30 | 0.49 ± 0.02 | 0.49 | 22.857 | b | w |
| *Pterostichus diligens* (Sturm, 1824) | 2 | 3 | 5.80 ± 0.49 | 5.88 | 0.47 ± 0.03 | 0.46 | 27.874 | b* | w |
| *Pterostichus madidus* (Fabricius, 1775) | 10 | 82 | 15.27 ± 0.63 | 15.26 | 0.49 ± 0.02 | 0.50 | 18.803 | b | d |
| *Pterostichus melanarius* (Illiger, 1798) | 7 | 9 | 15.89 ± 1.17 | 15.82 | 0.49 ± 0.03 | 0.50 | 26.153 | b* | d |
| *Pterostichus niger* (Schaller, 1783) | 4 | 9 | 18.35 ± 1.03 | 18.62 | 0.59 ± 0.02 | 0.58 | 31.451 | m | w |
| *Pterostichus vernalis* (Panzer, 1796) | 2 | 3 | 7.14 ± 0.28 | 7.10 | 0.48 ± 0.04 | 0.49 | 24.892 | m* | w |
